# A novel all‐arthroscopic technique for biomaterials insertion and positioning on shoulder rotator cuff: Preliminary insights and experience on a cohort of 34 patients

**DOI:** 10.1002/jeo2.70709

**Published:** 2026-06-16

**Authors:** Enrico Guerra, Fabio Tortorella, Eleonora Villari, Valentina Brunello, Matilde Tschon, Marco Cavallo

**Affiliations:** ^1^ Shoulder and Elbow Surgery IRCCS Istituto Ortopedico Rizzoli Bologna Italy; ^2^ Surgical Sciences and Technologies IRCCS Istituto Ortopedico Rizzoli Bologna Italy

**Keywords:** arthroscopy, augmentation, biomaterial, case series, rotator cuff repair

## Abstract

**Purpose:**

Rotator cuff repair is associated with a high risk of re‐tear, especially for large or chronic lesions. Although biological augmentation using grafts has been proposed for improving tendon healing, graft insertion and positioning remain technically challenging and frequently necessitate specialised equipment or graft‐specific techniques. This study describes a simple, reproducible and fully arthroscopic surgical technique for positioning and fixing different types of biomaterial graft on the rotator cuff, independently of graft type and prior anchor placement.

**Methods:**

A case series of 34 patients is presented. They are operated with a novel fully arthroscopic technique for preparing, inserting, positioning and fixing a decellularized human dermal graft on the rotator cuff. The procedure uses only arthroscopic portals and cannulas without dedicated instrumentation. The graft is prepared with colour‐coded sutures and tapes to indicate orientation, passed through the repaired cuff using non‐resorbable sutures and positioned under direct arthroscopic visualisation. Medial fixation is achieved through trans‐tendinous sutures, while lateral fixation is performed using knotless anchors placed on the greater tuberosity. Clinical evaluation is performed with Constant‐Murley and Patient Acceptable Symptomatic State (PASS) scores preoperative and at 6 months of follow‐up.

**Results:**

The described technique enables precise positioning of the graft over the repaired rotator cuff. Graft orientation, tensioning and greater tuberosity coverage can be adjusted independently of previous anchor placement. The method is compatible with different biomaterials and enables graft insertion without torsion, excessive tension or the need for mini‐open approaches or graft‐specific devices. No major or minor complication, and no graft dislocation has been reported at 6 months of follow‐up. Postoperative Constant‐Murley and PASS scores have significantly been improved from baseline.

**Conclusions:**

The novel, all‐arthroscopic technique provides a safe, versatile and reproducible method for augmenting the rotator cuff with biomaterial grafts, reporting good preliminary early‐term clinical results.

**Level of Evidence:**

Level IV, case series with no comparison group.

## Graphical abstract

A fully arthroscopic graft positioning method for the rotator cuff repair allows independent tensioning and fixation, avoids graft‐specific devices, and yields significant early clinical improvement without complications.

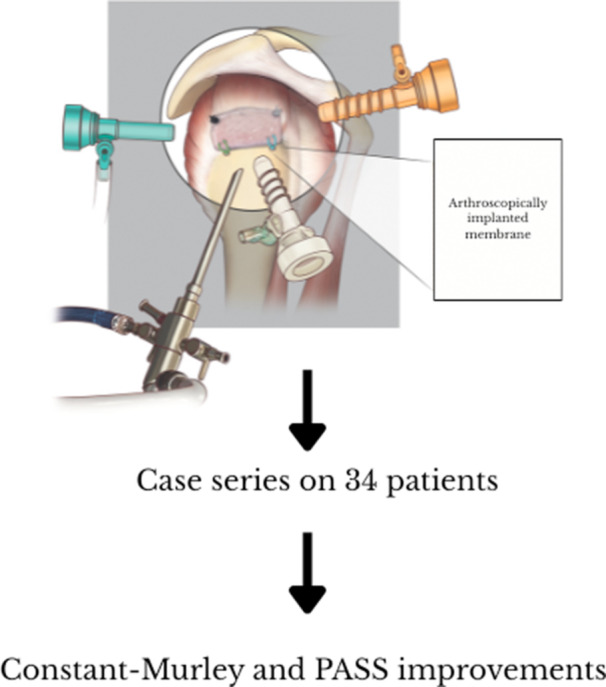

**Abbreviations**
ADLp: anterior distal lateral portal
ASp: antero‐superior portal
PDLp: posterior distal lateral portal
PLp: proximal lateral portal
Pp: posterior portal
RCR: rotator cuff repair

## INTRODUCTION

Arthroscopic rotator cuff repair (RCR) is one of the most commonly performed orthopaedic surgical procedures and typically resulting in improved shoulder function and high levels of patient satisfaction [18]. Nevertheless, one of the possible long‐term complications is re‐tear. Several factors have been shown to increase this risk, including health behaviours, such as smoking or alcohol use, patient demographics, shoulder anatomy, comorbidities, tissue quality and the original size of the rotator cuff tear [17, 20]. According to literature, the re‐tear rate following RCR can reach 40% for small to medium tears and 94% for large and chronic tears [16, 21]. Over time, various surgical procedures, such as revision RCR, superior capsular reconstruction, interposition grafting, tendon transfer and reverse total shoulder replacement, have been proposed for re‐tear cases with positive outcomes. Recently, attention has been turned to rotator cuff augmentation with different biomaterials [3].

Many studies have focused attention on an arthroscopically applied soft‐tissue allograft augmentation in cases of cuff tendinosis without rupture or following primary RCR, based on the hypothesis that strengthening the cuff will improve healing and subsequently decrease tear or re‐tear rate [1, 8]. These biomaterials can also be employed as a bridge to fill the gap between the cuff and the greater tuberosity in non‐complete repair of the cuff and are mostly composed of connective tissue with human, equine or porcine origin [4]. Regardless of the type of material used and the clinical outcomes, the positioning of a graft on the rotator cuff is a complex procedure without a dedicated insertion tool [28]. Several techniques have been proposed [7, 13, 25, 26], each dedicated to a particular biomaterial. Excellent previous results have been obtained by implanting acellular dermal graft for RCR, but without a specific and all‐arthroscopic surgical technique [24]. Moreover, a simple and reproducible technique, not related to a specific graft, has not yet been reported. The aim is to develop a full arthroscopic surgical technique that allows the positioning of a rotator cuff augmentation graft in the shoulder. The hypothesis is that the developed all‐arthroscopic technique would allow a graft positioning onto a rotator cuff without the necessity of a dedicated insertion tool. The safety of the technique is evaluated in a cohort of 34 patients.

## MATERIALS AND METHODS

With the aim to confirm the validity of this fully arthroscopic surgical technique, the experience on 34 patients affected by large‐to‐massive rotator cuff lesions was reported. The patient population belonged to an authorized and registered protocol (CE AVEC 154/2023/Sper/IOR). The study was a prospective consecutive case series.

Inclusion criteria were:

- Male or female patients between 18 and 65 years.
- Large‐to‐massive rotator cuff tear involving supraspinatus and infraspinatus tendons.
- Tendon retraction ≤ Thomazeau 3.
- Fatty infiltration ≤ Goutallier 3 at pre‐operative MRI.
- Possibility to obtain tendon reduction at footprint and complete humeral head coverage at the end of the RCR.
- Patients' ability and consent to participate in clinical and radiological follow‐up.

Exclusion criteria were:

- Shoulder osteoarthritis.
- Adhesive capsulitis.
- Symptomatic acromioclavicular arthritis.
- Previous RCR in the affected shoulder.
- Current or past haematological disorders.
- Chronic steroid use or comorbidities affecting healing.
- Patients with malignancy, rheumatic diseases or uncompensated endocrine diseases.
- Patients with alcohol or drug abuse.
- Pregnancy and lactation.
- Inability to cope with postoperative rehabilitation regimen.

Patients were recruited and treated between December 2023 and July 2025: preoperatively, patients were evaluated on an outpatient basis and by MRI imaging. Thirty‐eight patients met the inclusion criteria; during arthroscopy, four patients had irreparable lesions and were then excluded from the case series. Then, 34 patients affected by complete supraspinatus or infraspinatus lesions with no signs of muscle atrophy were operated. The surgical technique, here described, involved the RCR and the arthroscopic augmentation of the lesions by implanting a decellularized dermal allograft. Postoperatively, all patients followed the same rehabilitative protocol consisting of 1 month of sling abduction without movements, followed by passive movements for 2 weeks and active exercises for 6 weeks. The full recovery of daily and working activities were allowed three months after surgery. The patient demographics, including age, gender, involved tendons and side of pathology were recorded. Clinical outcomes were measured by the Constant‐Murley and Patient Acceptable Symptomatic State (PASS) scores at baseline and at 6‐month follow‐up period.

### Surgical technique

Patients were positioned in the beach chair position.

#### Portals set up

**Figure 1:**
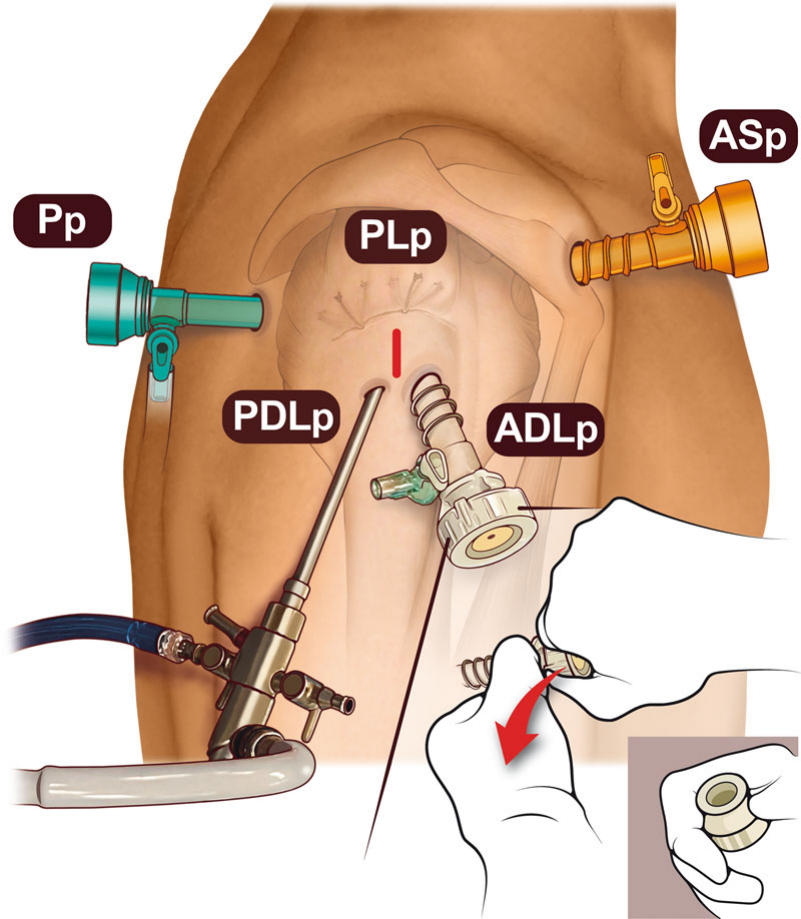
Portals set up: posterior portal (Pp), antero‐superior portal (ASp), proximal lateral portal (PLp), anterior distal lateral portal (ADLp) and posterior distal lateral portal (PDLp).

The working position to allow RCR and consequent graft implantation started with standard posterior (Pp) and antero‐superior portals (ASp). A threaded working cannula of 8 mm diameter was positioned in the ASp, and a smaller 7 mm cannula was positioned in the Pp (Figure 1).

Three different additional lateral portals were then needed:

– A proximal lateral portal (PLp) is used for lateral graft fixation. This portal was made in the direction of the dead man angle on the greater tuberosity and was the same used for anchor insertions.
– Two distal lateral portals:

- One anterior (anterior distal lateral portal, ADLp) for graft insertion and lateral fixation.
- One posterior (posterior distal lateral portal, PDLp) is used as a view portal.

A full‐threaded 8.5 mm cannula with a removable top was then positioned in the ADLp.

#### Arthroscopic RCR

**Figure 2:**
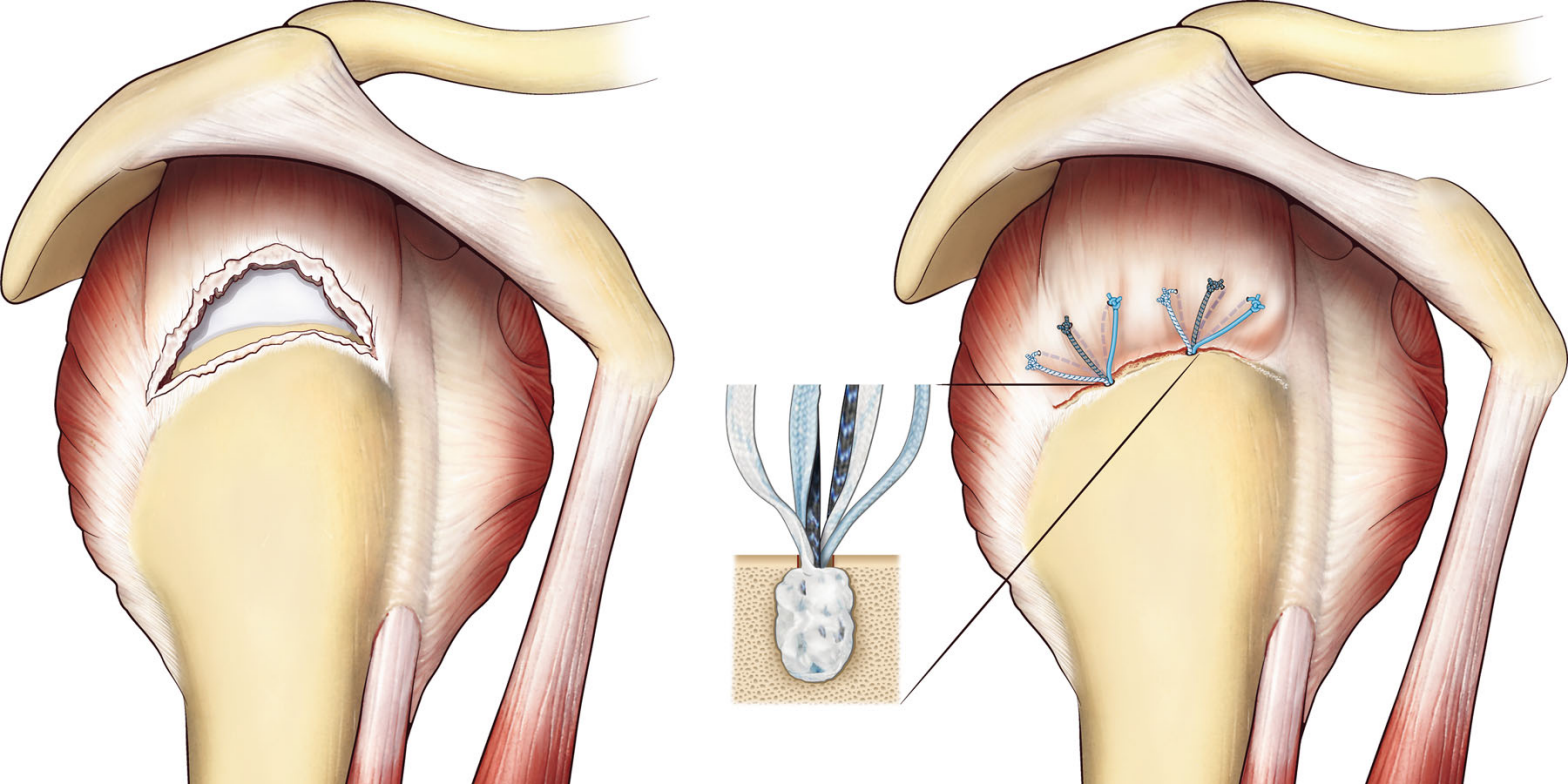
Rotator cuff tear repair with two anchors.

A standard shoulder arthroscopy was performed, with RCR complete repair in all cases (Figure 2). Wide bursectomy was necessary, and the greater tuberosity should be debrided for graft positioning.

#### Graft preparation

The area to be grafted was measured by an arthroscopic probe through ASp and ADLp, which permitted cutting or shaping the patch accordingly.

**Figure 3:**
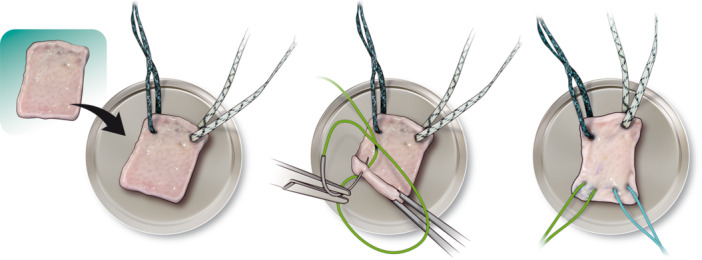
Graft preparation using two tapes for the posteromedial and anteromedial corners and two No. 5 non‐resorbable mattress sutures for the posterolateral and anterolateral corners.

Firstly, anterior, posterior, medial and lateral sides of the graft were identified. Two tapes of a different colour were stitched close to the posteromedial and anteromedial corners. Dynatape (DePuy Synthes) was preferred, which has a low cutting effect and the benefit of improving tension after the surgery. Then, the posterolateral and anterolateral corners were stitched with two mattress sutures, using No. 5 non‐resorbable sutures. The darker tapes/sutures were conventionally positioned posteriorly (Figure 3).

#### Graft positioning

**Figure 4:**
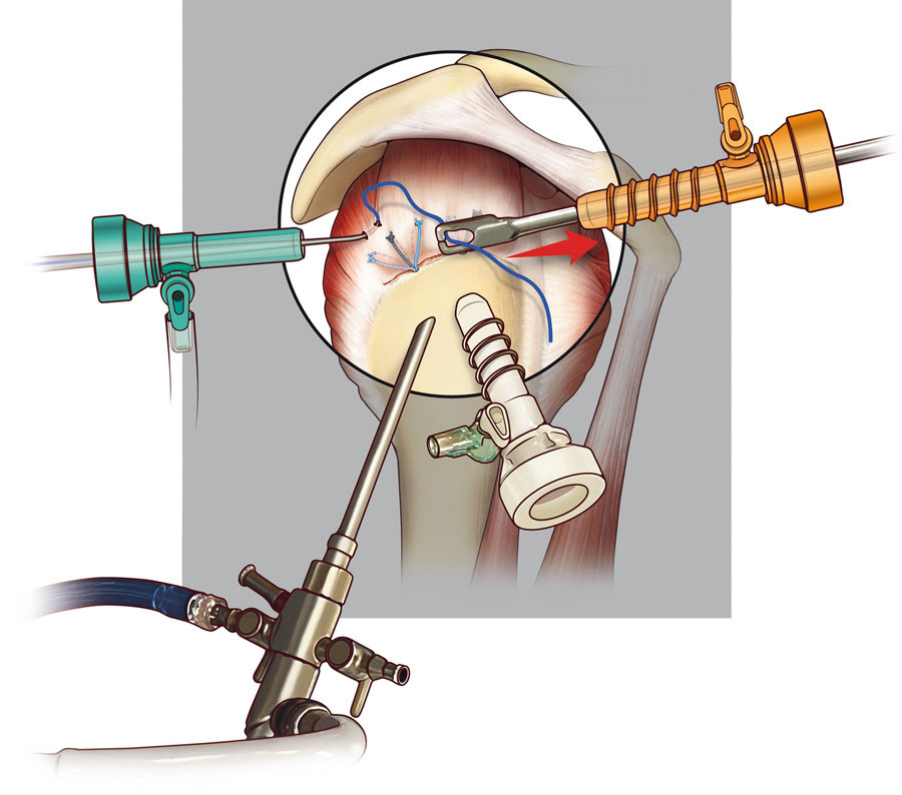
A first suture was shuttled in the postero‐medial side of the cuff and retrieved from the ASp. ASp, antero‐superior portal.

A first absorbable No. 1 suture was shuttled through the posterior cannula (Smith & Nephew) in the postero‐medial side of the cuff (infraspinatus muscle) by a straight cuff instrument and then retrieved from the ASp (Figure 4).

**Figure 5:**
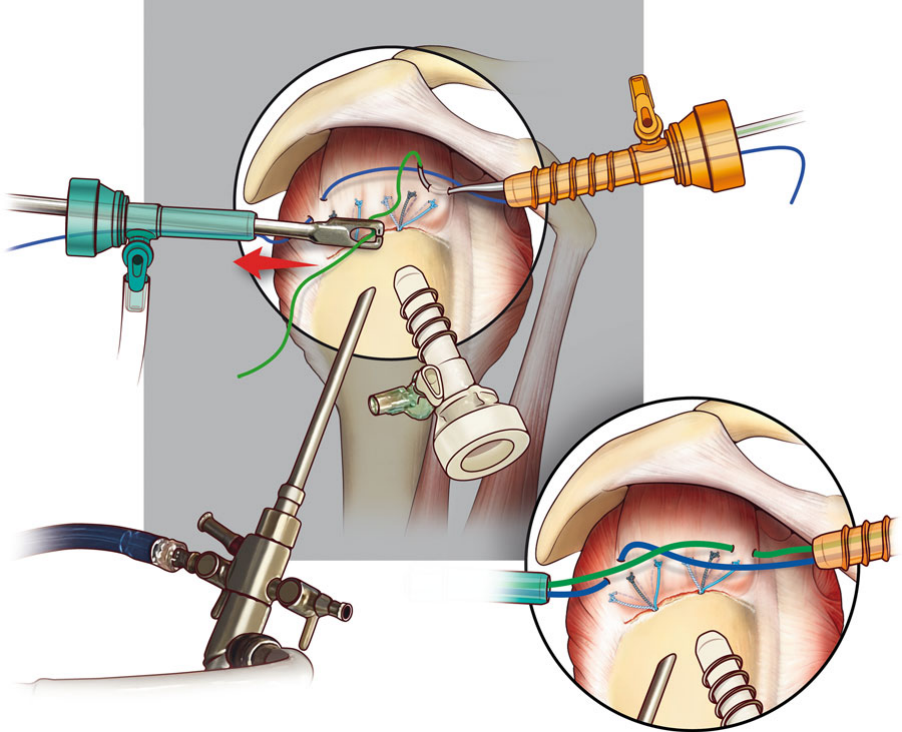
A second suture was shuttled in the anterior side of the cuff and retrieved from the Pp. Pp, posterior portal.

A second suture was placed in the anterior side (supraspinatus muscle) by a curved suture jig and retrieved from the Pp (Figure 5).

**Figure 6:**
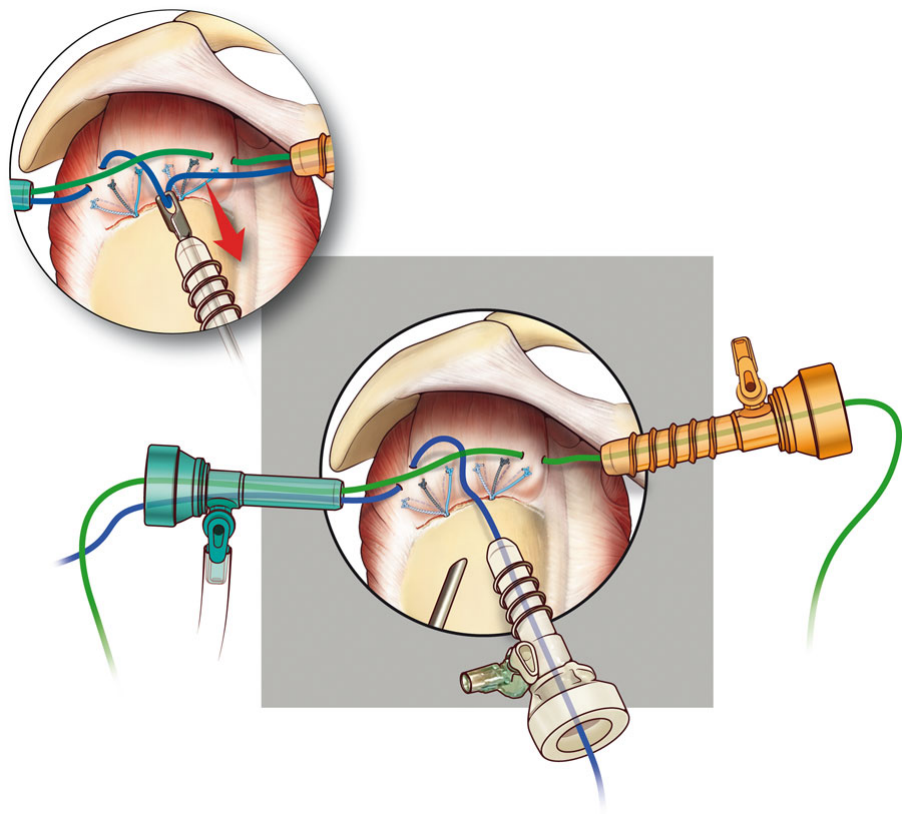
No. 1 sutures were replaced by No. 2 non‐resorbable sutures of two different colours. The anterior edge of the posterior stitch was retrieved from ADLp for further graft insertion. ADLp, anterior distal lateral portal.

The No. 1 monofilament sutures were then replaced by two stronger No. 2 non‐resorbable sutures, which needed to be of two different colours to be easily distinguished (Figure 6). This step was required because the No. 1 monofilament sutures used for cuff passage with the angulated tools would break due to the traction for graft insertion. To allow the graft to lay down after insertion, the gap between anterior and posterior stitches must be greater than the graft width.

**Figure 7:**
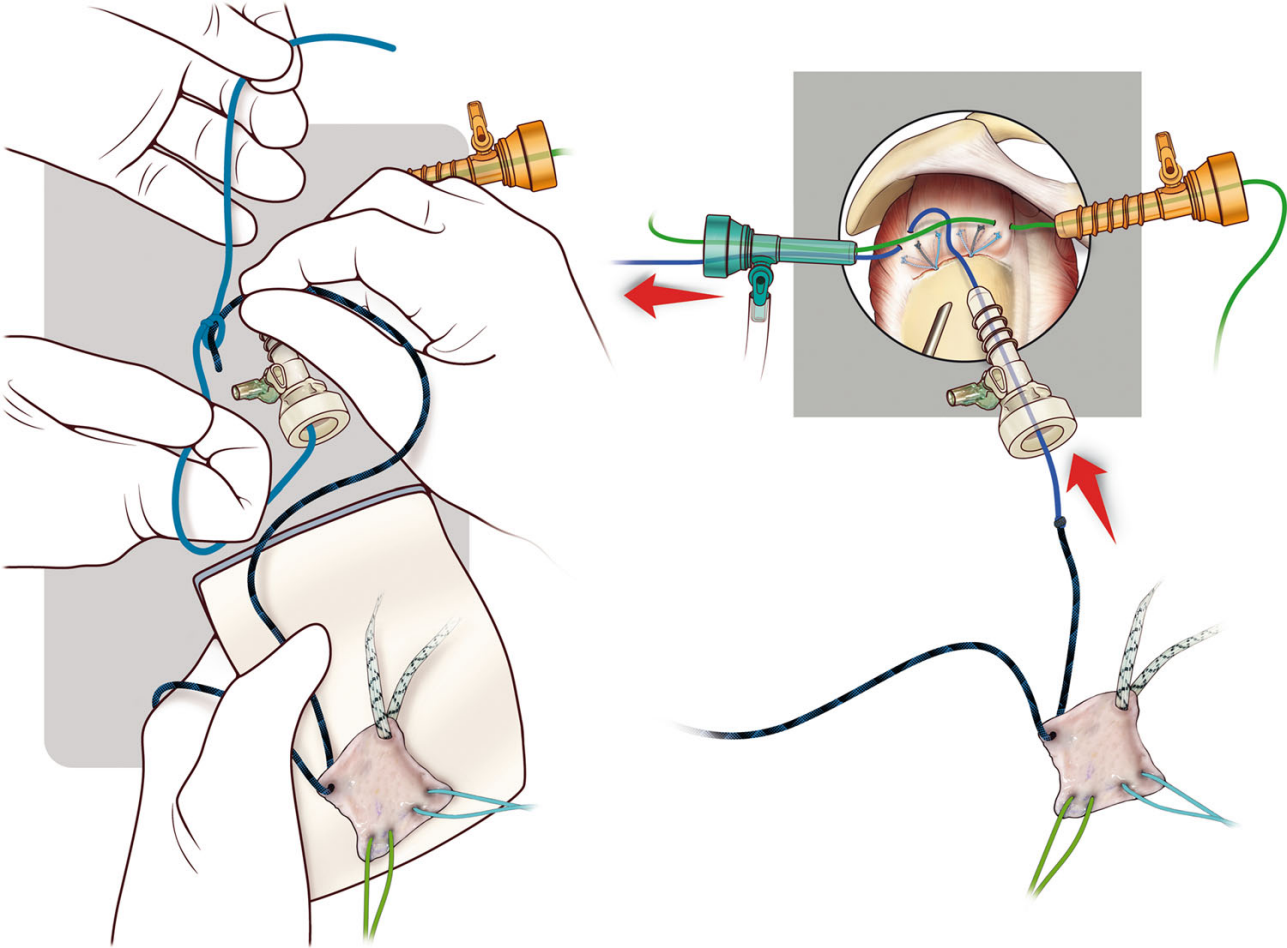
The posterior No. 2 suture was temporarily tied to the inferior part of the suture‐tape from the posterolateral corner of the dermal graft to prepare for shuttling the tape.

The No. 2 stitches were used to shuttle the two tapes of the graft through the cuff. The top of the lateral cannula (CONMED Co) was then removed, and the inferior edge of the tape was passed by, retrieving the posterior Ti‐cron through ADLp starting from the posterior tape (Figure 7).

A large diameter cannula, at least 8 mm, with removable diaphragm was selected: a standard cannula with fluid‐stop diaphragm would damage the graft during its insertion. This type of cannula allowed to maintain the distension fluid pressure while the diaphragm was on (before and after graft insertion) and did not interfere with the graft during its insertion.

**Figure 8:**
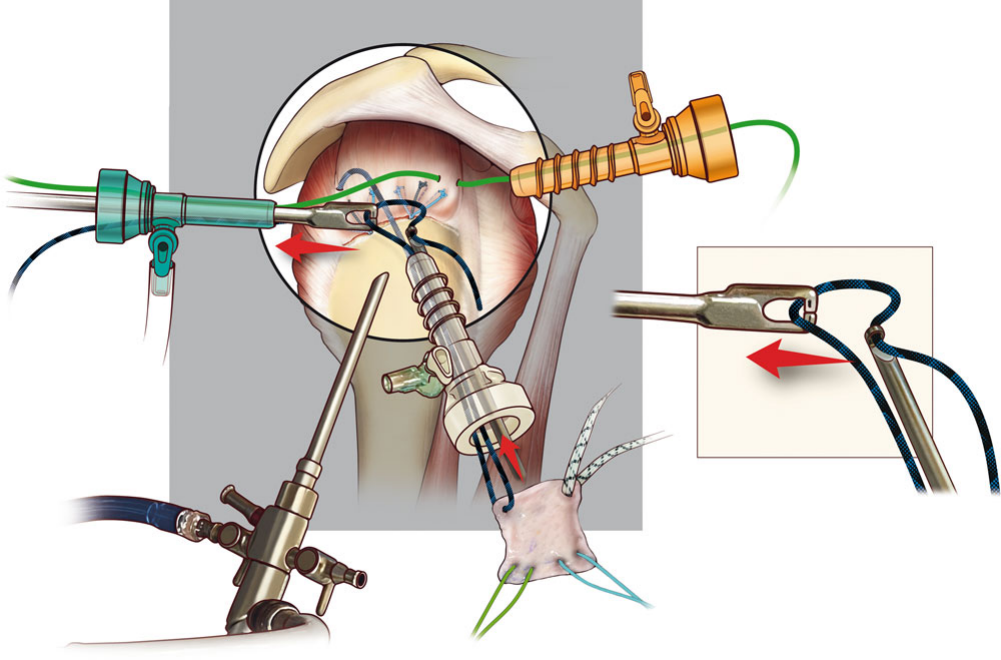
The superior edge of the posterior tape was passed with a knot pusher from ADLp and retrieved from Pp. ADLp, anterior distal lateral portal; Pp, posterior portal.

The superior edge of the same posterior tape was then shuttled with a knot pusher through the ADLp and retrieved from Pp (Figure 8).

Following the same steps, the anterior tape was placed, using the anterior shuttle suture.

**Figure 9:**
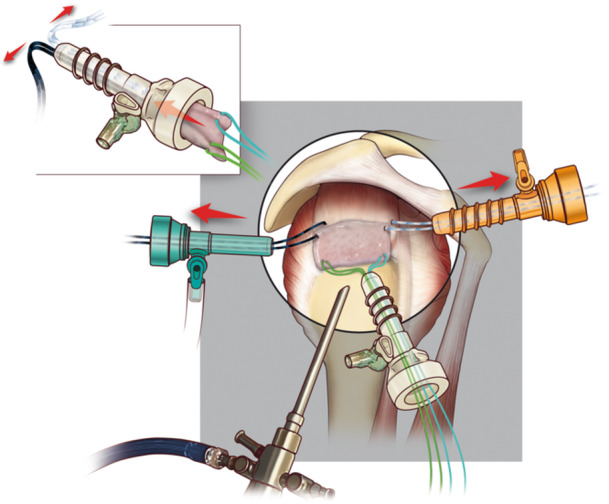
The graft was carried through the lateral cannula by gentle traction and placed covering the repaired cuff.

The graft was ready to be carried on through the lateral cannula with a gentle traction on both tapes, under direct view from PDLp. Following these passages, the patch was placed to cover the repaired cuff. It was crucial to avoid graft torsions or stitch loops (Figure 9).

**Figure 10:**
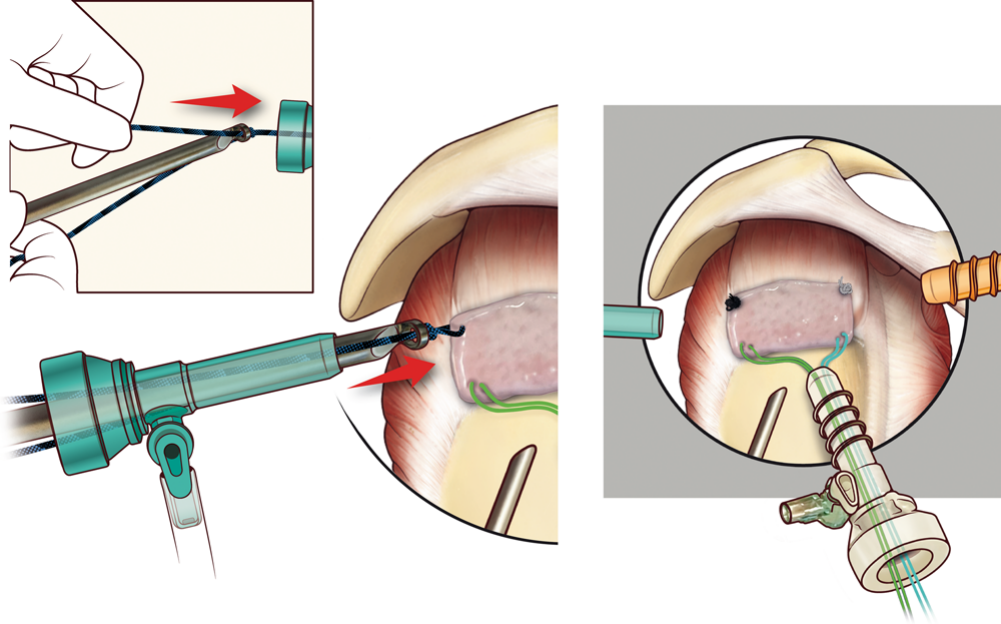
Tapes tied with simple knots.

Once the correct position of the graft was confirmed, tape sutures were tied with simple knots using an arthroscopic knot pusher (Figure 10).

**Figure 11:**
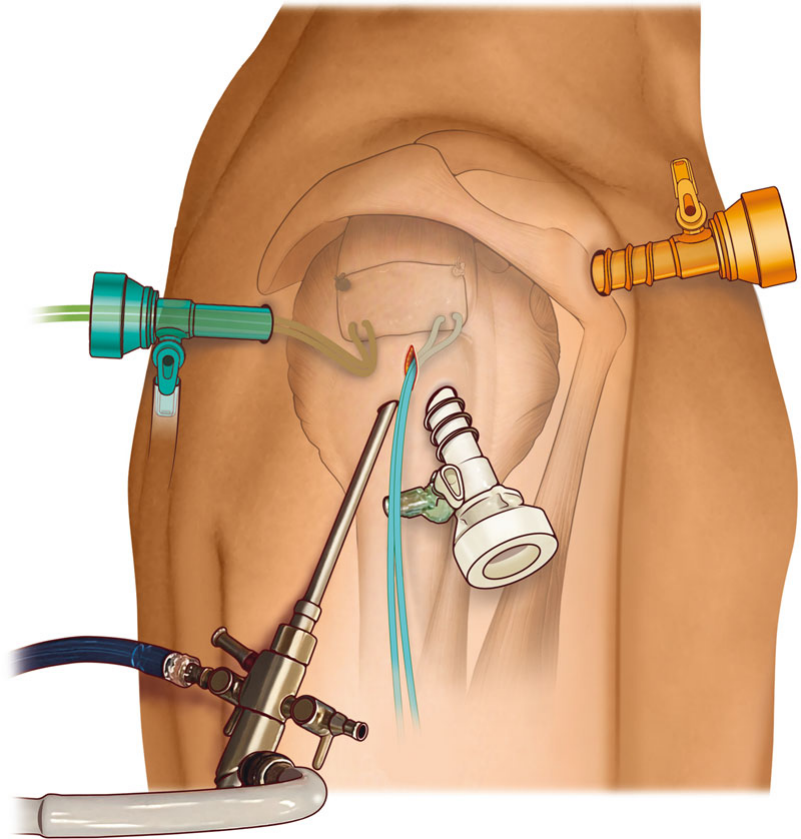
Lateral sutures identification and isolation.

For lateral dermal graft fixation, the two pairs of lateral sutures had to be identified and isolated. The posterolateral sutures (darker) were retrieved from the Pp while the anterolateral sutures (lighter) were caught through the PLp (Figure 11).

**Figure 12:**
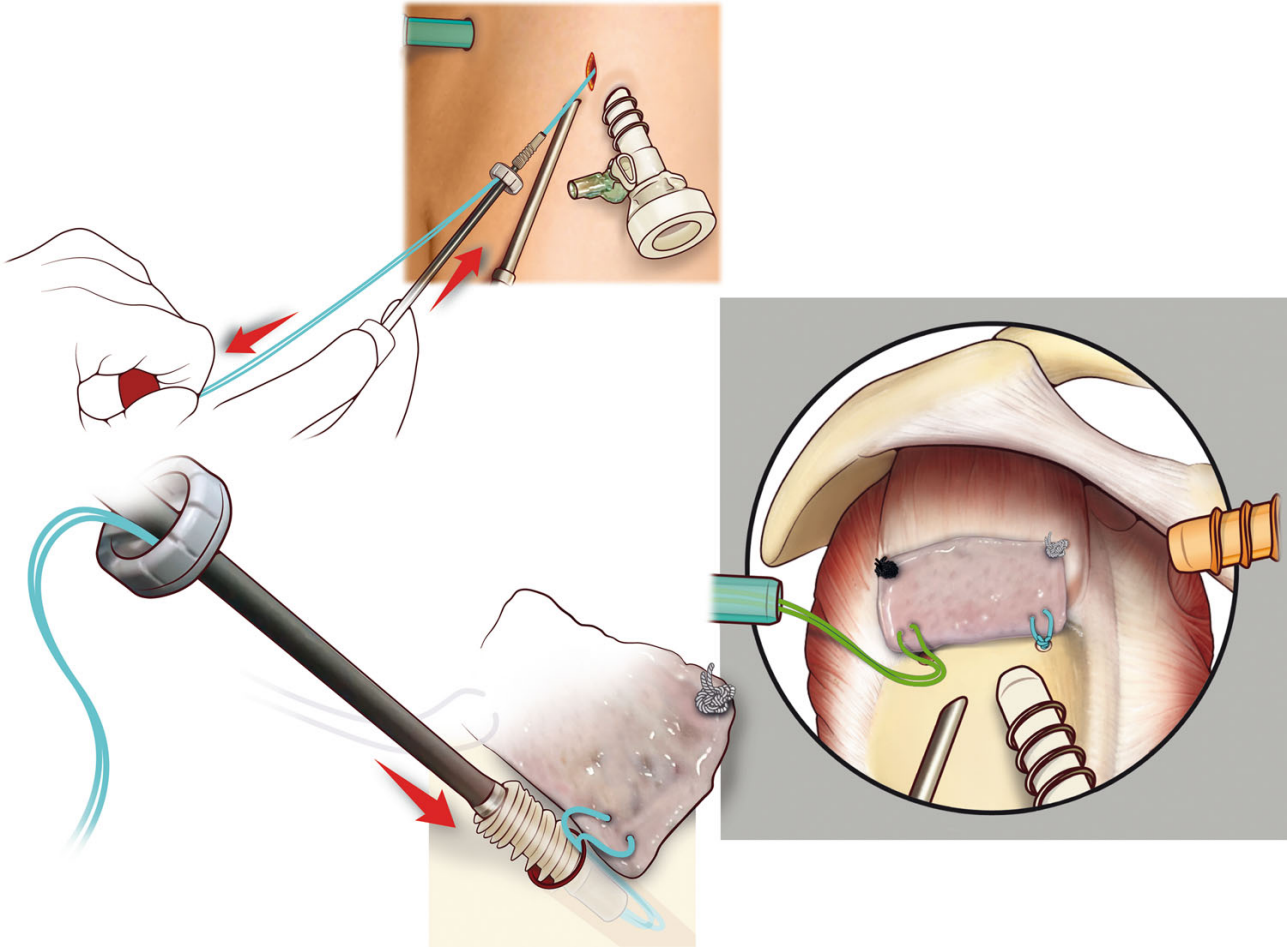
Knotless anchor insertion through the PLp to tie the anterolateral corner of the dermal patch. PLp, proximal lateral portal.

A knotless absorbable anchor (Healix Advance Knotless; Depuy Synthes) was inserted through the PLp, at the anterolateral portion of the greater tuberosity, to secure the anterolateral corner of the dermal patch. The two ends of the anterolateral suture were used to make sure that the graft was neither over nor under‐tensioned (Figure 12).

**Figure 13:**
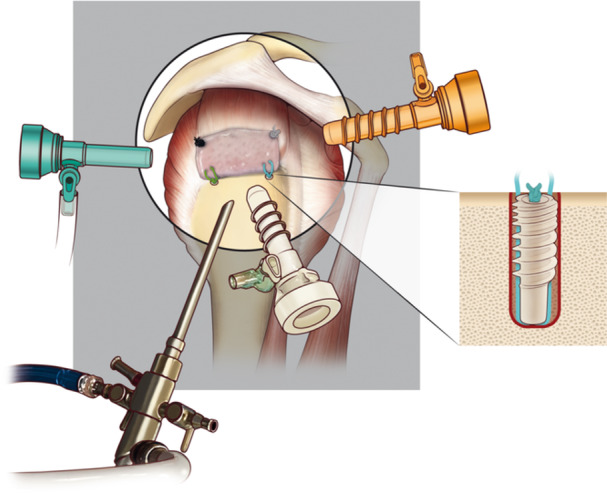
Second anchor placement on the postero‐lateral surface of the greater tuberosity.

Once the first anchor was placed in the anterolateral portion of the greater tuberosity, the posterior sutures were retrieved through the PLp and fixed by a second knotless anchor. The anchor was positioned posteriorly to the first one, on the postero‐lateral surface of the greater tuberosity (Figure 13).

If the graft position was acceptable and big enough to cover the entire greater tuberosity surface, the knotless anchor fixation of the lateral sutures could be performed directly through the lateral cannula in the ADLp.

The video (Video S1) provides a comprehensive overview of the surgical procedure, illustrating each step involved in the positioning of the biomaterial.

### Statistical analysis

Data were analysed with GraphPad Prism (v 10.4.2) and checked for normality by the Kolmogorov–Smirnov test; since data were not normally distributed, the Wilcoxon matched pairs signed rank test was applied to Contant‐Murley results to detect significant differences between baseline and 6‐month follow‐up. PASS values were analysed with Fisher's exact test. The minimal clinically important difference (MCID) for the Constant‐Murley score was defined *a priori* as an improvement of ≥10.4 points, consistent with thresholds reported for clinically meaningful change in rotator cuff–related shoulder conditions [14]. Each patient was classified as having achieved MCID based on the individual change from baseline to 6 months of follow‐up. The PASS was assessed using a dichotomous patient‐reported item (‘YES/NO’). The proportion of patients reporting PASS at 6‐month follow‐up was calculated. Exact binomial 95% confidence intervals were computed for both MCID and PASS proportions.

## RESULTS

This technique was developed with the specific aim of allowing graft positioning on a RCR, regardless of the different types of graft and with no need for specifically designed equipment.

The mean age of the cohort was 54.0 ± 6.3 years, 16 females and 18 males; lesions affected the supraspinatus tendon for all patients, and two patients reported a lesion also in the infraspinatus tendon. The sites of lesions were mostly on the right side (24/34). Table 1 shows the baseline characteristics of included patients.

**Table 1:** Baseline characteristics of the included patients.

Baseline characteristics	Frequency (*N*)
Male	53% (18)
Female	47% (16)
Lesion site (right)	71% (24)
Lesion site (left)	29% (10)

**Figure 14:**
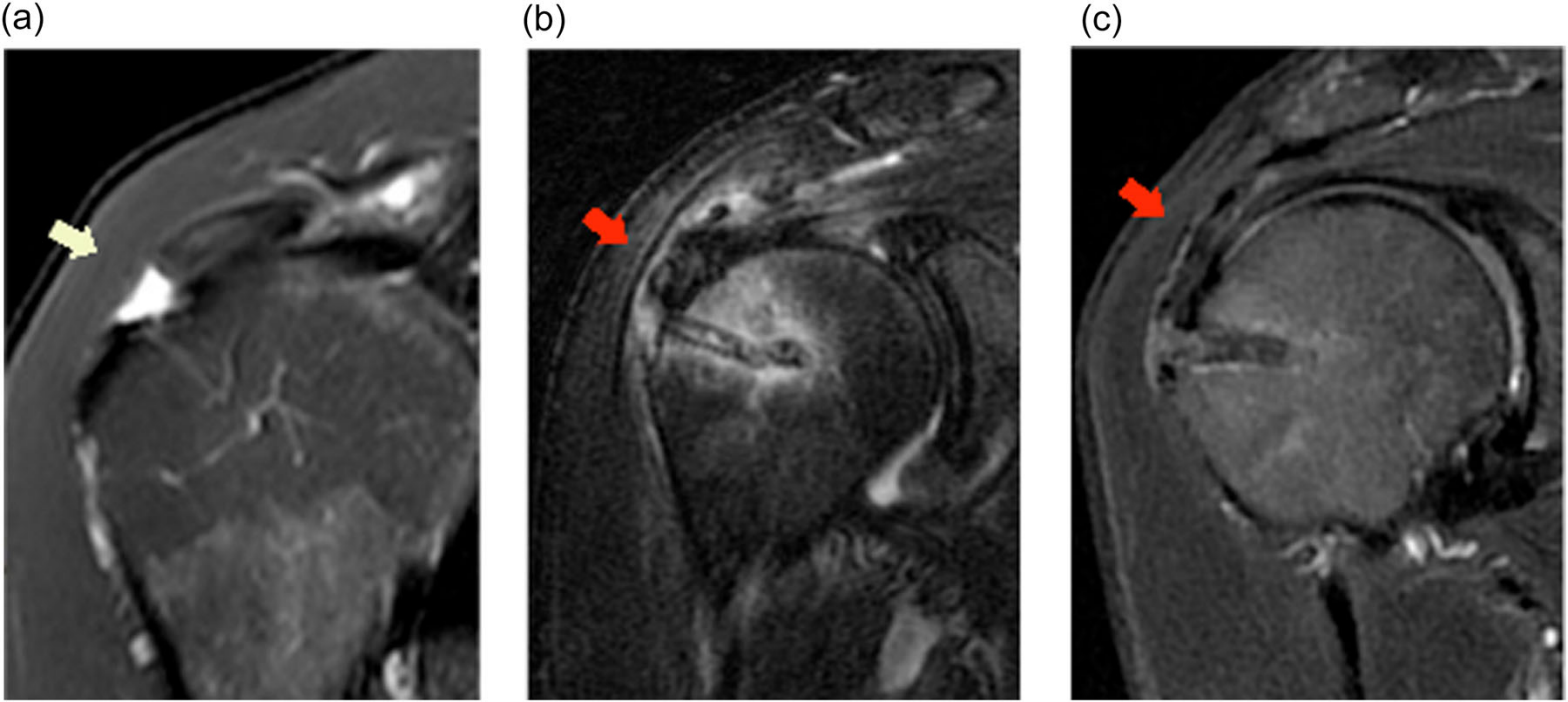
MRI imaging. (a) Preoperative MRI of the patient shoulder with a complete supraspinatus tear with minimal retraction (yellow arrow); (b) 2‐day postoperative MRI of the same patient showing the supraspinatus repair with the decellularized dermal graft lining on the top (red arrow); (c) 6‐month postoperative MRI of the same patient showing the repaired tendon with the decellularized dermal graft still present (red arrow) and partially integrated with native tendon. MRI, magnetic resonance imaging.

MRI evaluation was performed at 6 months in three cases, in order to assess the presence of the graft: in Figure 14 preoperative, 2‐day and 6‐month postoperative MRI images were reported, highlighting the correct positioning of the decellularized graft and its partial integration with the native tendon after 6 months from surgery. The Constant‐Murley score showed a significant postoperative improvement, increasing from 60.6 ± 17.0 at baseline to 85.4 ± 14.1 at 6‐month follow‐up. The Wilcoxon signed‐rank test confirmed a highly statistically significant change (*p* < 0.001).

Using an MCID threshold of ≥10.4 points, 25 of 34 patients (73.5%; 95% confidence interval [CI]: 55.6%−87.1%) achieved a clinically meaningful improvement. The same proportion of patients (73.5%; 95% CI: 55.6%−87.1%) reported an acceptable symptomatic state (PASS = YES) at 6 months and significantly improved from baseline (*p* = 0.0136) (Table 2).

**Table 2:** Clinical outcome results.

Clinical scores	Baseline	6 Months FU	*p* value
Constant‐Murley	60.6 ± 17.1	85.4 ± 14.1	<0.0001
PASS (%YES)	41	74	0.0136

*Note*: Constant‐Murley data were expressed as mean ± SD. Wilcoxon matched pairs signed rank test was used to detect significant differences in the Constant‐Murley score results and Fisher's exact test in the PASS results. Constant‐Murley and PASS scores were recorded at baseline and at 6 months and statistical analysis results.

Abbreviations: FU, follow‐up; PASS, Patient Acceptable Symptomatic State; SD, standard deviation.

## DISCUSSION

The positioning of a graft as augmentation or bridging in cases of incomplete repair following arthroscopic RCR is a topic of debate within the scientific community in recent years. Many studies describe techniques for graft insertion by mini‐open approach [5, 6, 10]. The aim of the study is to develop and describe a safe and easily reproducible all‐arthroscopic technique for positioning a graft on a rotator cuff. Besides the early‐term follow‐up time of 6 months, the developed technique results in good and improved clinical outcomes with clinical relevance, as highlighted by the MCID.

Nowadays, only a few companies offer biomaterials together with dedicated insertion tools and related surgical procedures [23, 28], leading to the necessity of arthroscopic techniques for these graft options. On the other hand, a huge number of biomaterials are proposed to augment or bridge rotator cuff lesions, often positioned with mini‐open technique or, in some cases, with arthroscopic technique. Some authors describe an interesting technique that uses medial anchor sutures to fix the biomaterial [27], or the autograft made by the long head of the biceps [15, 19]. However, the major limit is that the surgeon must decide from the beginning of the procedure whether a graft will be placed and, consequently, insert the anchor. This issue constrains the shape and size of the graft according to the position of the anchors. In a similar study, the scaffold is placed on the articular side of the rotator cuff [29]. In this scenario as well, the surgeon has to determine where to place the graft before the RCR. Another option for graft placement is the ‘Canova’ technique: a standard RCR with a double‐row construct is performed, and then a graft is placed [12]. According to the same authors, this approach is not ideal for the rotator cuff since it cannot be mobilized or has too much tension after repair, but it does allow the surgeon to easily augment the repair as needed without extra anchors or knots. Furthermore, the use of the anchors for both RCR and graft fixation makes it impossible to employ larger grafts or cover different portions of the rotator cuff, including bridging the non‐repaired portion of the tendon.

The presented technique allows the surgeon to decide whether to put a graft or not when the RCR is completed, with the possibility to choose its position independently of the previous anchor placement on the rotator cuff and even to cover the greater tuberosity. This technique may be used not only after RCR but can also be considered as an augment patch in case of cuff tendinosis, since it does not rely on medial anchors for its fixation.

The graft used for this study is a homologous decellularized derma, provided by Emilia Romagna Regional Skin Bank. This biomaterial is chosen because it combines excellent biological and mechanical properties and shows good clinical outcomes in RCR [2, 9, 11, 24].

Regarding the fixation technique, in several studies, the double row technique is used to provide stronger fixation of the rotator cuff at the greater tuberosity than single row technique, but without clinically relevant differences [22]. In the present study, the single row repair is used to leave the graft positioning independent from the cuff repair, and to preserve the graft from multiple sutures that could possibly obstruct the tendon vascularization, graft integration and tendon healing. In fact, a double‐row technique would have required four anchors for the cuff repair plus the two knotless anchors for lateral graft fixation, with the risk of lack of space in greater tuberosity, especially in small patients.

## CONCLUSION

This work aims to present a novel, simple, reproducible and safe technique for biomaterial insertion, positioning and securing into a rotator cuff. Our experience shows preliminary insights about safety and reproducibility on a cohort of 34 patients implanted with a decellularized dermal allograft with no intraoperative major complication and significantly improved Constant‐Murley and PASS scores at 6 months. Despite the short follow‐up period, the steps described allow any biomaterial of the surgeon's choice to be positioned, regardless of the previous RCR repair and anchor positioning. This allows the graft to be positioned where preferred. This technique can also be used on a degenerated rotator cuff, as biological augmentation without RCR, or in the case of graft use as bridging in incomplete RCR in massive tears.

## AUTHOR CONTRIBUTIONS

*Conceptualization*: Enrico Guerra and Marco Cavallo. *Methodology*: Enrico Guerra and Marco Cavallo. *Formal analysis and investigation*: Fabio Tortorella, Eleonora Villari and Valentina Brunello. *Writing—original draft preparation*: Fabio Tortorella, Marco Cavallo and Eleonora Villari. *Writing—review and editing*: Matilde Tschon, Valentina Brunello and Enrico Guerra. *Funding acquisition*: Matilde Tschon. *Resources*: Enrico Guerra and Matilde Tschon. *Supervision*: Enrico Guerra and Matilde Tschon.

## CONFLICT OF INTEREST STATEMENT

The authors declare no conflicts of interest.

## ETHICS STATEMENT

This study was performed in line with the principles of the Declaration of Helsinki. Approval was granted by the Ethics Committee of Area Vasta Emilia Centro (CE AVEC154/2023/SPER/IOR, March 23, 2023). Informed consents to participate have been obtained from participants.

## Supporting information


**ESM_1** Video animation of the main steps of the surgical technique for the biomaterial′s positioning.

RAW DATA3.

## Data Availability

The data that support the findings of this study are available in the supporting information of this article.
